# Rod-Shaped NanoZnTPyP Paper-Based Sensor for Visual Detection of Dopamine in Human Plasma

**DOI:** 10.1155/jamc/9981628

**Published:** 2025-04-14

**Authors:** Linlin Yin, Yuyu Du, Miaohua Ge, Xiang Zhang, Xinyi Du, Xiaoqiong Wu

**Affiliations:** ^1^Jiaxing Center for Disease Control and Prevention, Jiaxing, China; ^2^Jiaxing Jiayuan Testing Technology Service Co., Ltd, Jiaxing, China

**Keywords:** CdTe quantum dots, dopamine, fluorescence sensor, paper-based sensor, rod-shaped nanoZnTPyP

## Abstract

Dopamine (DA) is a catecholamine neurotransmitter secreted by the human adrenal medulla and is related to many medical diseases. The rapid and sensitive detection of DA levels in physiological media is attracting attention. This paper has developed a fluorescence paper-based sensor using CdTe quantum dots (QDs)-rod nanozinc 5, 10, 15, 20-tetra (4-pyridyl)−21H-23H-porphine (nanoZnTPyP) for sensitive and visual detection of DA. After adding DA, the original quenching fluorescence of the CdTe QDs-rod nanoZnTPyP sensor was effectively restored. The detection mechanism may be that the oxidation of DA to the alkaline CdTe QDs-rod nanoZnTPyP solution produced DA-quinine, and the recovery of fluorescence was caused by the electronic effect of DA-quinine and rod-shaped nanoZnTPyP. The detection range is 0.5∼10 nmol/L, and the limit of detection (LOD) is 0.38 nmol/L (S/N = 3). The sensor system was used on paper device to detect significant changes in the fluorescent color of DA at different concentrations. In addition, this method has been successfully used for the determination of DA in human plasma. The sensor system is simple, easy to operate, and has high selectivity for possible DA interfering substances, which provided new ideas for detecting DA and Parkinson's disease, Alzheimer's disease, and other DA-related diseases.

## 1. Introduction

Dopamine (DA) is a catecholamine neurotransmitter secreted by the human adrenal medulla. Its concentration disorder is closely related to neurological diseases. Abnormal and insufficient DA concentrations often lead to a series of neurological diseases, such as hyperactivity disorder [1], Parkinson's disease [2], Alzheimer's disease [3] and schizophrenia [4]. Therefore, the diagnosis of such diseases requires a highly sensitive and accurately measured DA concentration. From the perspective of biomedicine and human health, a variety of methods for the determination of DA content have been developed in the past, including chromatography [5, 6], spectroscopy [7, 8], electrochemical [9, 10], and colorimetry [11], etc. Although chromatography has the advantages of high sensitivity and high accuracy, it usually relies on professionals and expensive instruments, which are time-consuming and consumable. Electrochemical has been proven to have higher sensitivity and selectivity, lower cost and simpler instruments. However, DA and ascorbic acid (AA) are oxidized at similar potentials, resulting in greater interference [12]. Spectral analyses mainly include fluorescence method and ultraviolet-visible (UV-Vis) spectrophotometry. Spectroscopy has strong specificity, strong timeliness, and large-scale detection ability. However, when using spectroscopy, it is necessary to ensure the stability of external or internal factors such as optical system parameters. Colorimetry has the advantages of simplicity, low cost and real-time detection. However, the use of colorimetry must ensure a low detection limit of the technology [13]. Therefore, it is urgent to develop simple, easy-to-operate and powerful anti-interference detection methods to accurately measure the level of DA.

Nanomaterials have small size effects, quantum size effects and dielectric limited domain effects, which give them unparalleled properties in mechanics, electricity, magnetism, thermal, optical, and chemical activities [14, 15]. Over the past few decades, many studies have focused on colloidal nanoparticles (NPs), which have become the most important components of sensitive optical nanosensors [16]. Fluorescent nanomaterials including quantum dots (QDs), metal nanoclusters (MNCs) and carbon dots (CDs) have been reported to detect DA [17, 18]. In addition, porphyrins have been widely used in the detection of pesticides [19], biological small molecules [20] and metal ions [21]. The nanocharacterization of porphyrins can be used to improve their optical properties and affinity. The “Turn-off-on” model based on QDs and nanoporphyrins has been successfully applied to amino acid [22], pesticide residue [23, 24], DNA research [25], and other fields. The application of QDs and nanoporphyrins has shown good development prospects in the field of sensors. Moreover, the paper sensor device method has great advantages in sensor analysis due to its simple operation and easy observation of color change [26]. However, methods based on the high sensitivity and selective detection of DA based on fluorescent paper-based sensor are rare.

In this study, we proposed a label-free DA fluorescence detection method based on water-soluble CdTe QDs and rod-shaped nanoZnTPyP. The fluorescence resonance energy transfer (FRET) between CdTe QDs and rod-shaped nanoZnTPyP caused the fluorescence quenching of CdTe QDs. DA was added to the alkaline CdTe-rod nanoZnTPyP solution and oxidized to produce DA-quinine. The electronic effect of DA quinine is due to the directional movement of electron clouds caused by the different electronegativity of DA quinine and rod shaped nanoZnTPyP induced the fluorescence recovery of CdTe QDs [27]. Based on the above reaction mechanism, a sensitive, simple, and low-cost method for detecting DA was developed by recording the fluorescence intensity of the CdTe-rod nanoZnTPyP system. This method has demonstrated sufficient stability and high accuracy in the detection of human plasma matrix samples. Moreover, the method further realized the rapid and visual detection and analysis of DA on the paper-based sensor.

## 2. Experimental Sections

### 2.1. Reagents and Instruments

DA hydrochloride was purchased from Shanghai Yuanye Biological Co., Ltd. (Shanghai, China). Cadmium chloride (CdCl_2_), sodium borohydride (NaBH_4_), acetone, hydrochloric acid, Dodecyl trimethylammonium bromide (DTAB) potassium chloride, sodium chloride, zinc chloride, calcium chloride, copper chloride, ferric chloride, barium carbonate, magnesium sulfate, nitric acid. Aluminum, uric acid, adrenaline, L-lactic acid, L-AA, catechol, β-phenethylamine and human plasma were all purchased from Sinopharm Chemical Reagent Co., Ltd. (Shanghai, China). 3-hydroxymethyl-methane (Tris) was supplied by Sigma-Aldrich. Sodium citrate, N-acetyl-L-cysteine (NAC) was supplied by Aladdin Biochemical Technology Co., Ltd. (Shanghai, China). Quantitative filter papers were obtained from Hangzhou Wohua Filter Paper Co., Ltd. (Zhejiang, China). Transmission electron microscope (TEM, TECNAI G2 20 S-TWIN, Oregon, USA) was used to characterize the morphology and particle size of CdTe QDs. UV-Vis spectra were recorded by using a UV-Vis Spectrophotometer (UV-1800 PC) (MaPADA, Shanghai, China). The fluorescence spectrum was obtained by scanning with an F-7000 fluorescence spectrophotometer (Hitachi High-Tech Company, Tokyo, Japan). Fourier transform infrared (FT-IR) spectra were measured by a Thermo Fisher Nicolet 6700-FT-IR spectrometer (Thermo Fisher Nicolet, Massachusetts, USA). An electrothermal constant temperature blast drying oven (Senxin DGG-9030B, Shanghai, China) was used for the synthesis experiment. The digital thermostatic water bath (XMTD-204) is owned by the shore Guorui Experimental Instrument Factory (Jintan, China). The CNC ultrasonic cleaner (KQ-500DE) was purchased from Ultrasonic Instrument Co., Ltd. (Kunshan, China). The laser cutting machine (XB-3020) was supplied by Xinbang Laser Equipment Co., Ltd. (Shandong, China). All photos were taken by the smartphone. Using Photoshop software, the obtained photos were simulated to extract RGB values.

### 2.2. Synthesis of CdTe QDs

CdTe QDs were synthesized by a reported method with minor modifications [23]. 0.0857 g of CdCl_2_ and 0.0735 g of NAC were weighed into a 100 mL round bottom flask, 30 mL of ultrapure water was added, stirred for 15 min in a magnetic stirrer, and the pH was adjusted to about 8.85 with 4 mol/L NaOH. It was filled with nitrogen in the ice bath. After that, 0.0216 g of sodium tellurite was weighed and dissolved in a small amount of water, added to the round bottom flask and stirred for 15 min. Weighed 0.0113 g of sodium borohydride dissolved in a small amount of water and quickly added to the round bottom flask, stirred for 15 min. Finally, it was poured into the reactor and reacted in the oven at 200°C for 50 min. Red CdTe QDs modified with NAC were obtained.

### 2.3. Synthesis of Rod-Shaped NanoZnTPyP

Rod-shaped nanoZnTPyP was synthesized by self-assembly [28]. An appropriate amount of ZnTPyP was dissolved in DMF solution (1.39 × 10^−3^ mol/L). DTAB (0.555 g) was dissolved in 90 mL of aqueous solution (1.8 × 10^−3^ mol/L), and 1 mL of ZnTPyP-DMF solution was added. The green transparent colloidal solution was obtained after high temperature microwave drying for 5∼8 min. The concentration of nanoZnTPyP was 1.5275 × 10^−5^ mol/L.

### 2.4. Fluorescence “Turn-Off-On” Analysis of DA Process

CdTe QDs solution (1.6063 × 10^−6^ mol/L) was placed in the cuvette, rod-shaped nanoZnTPyP with a concentration of 6.6375 × 10^−7^ mol/L and DA samples with different concentrations were added to the cuvette, and diluted with Tris-HCl buffer solution and reacted for 3 min. Then CdTe QDs (1.6063 × 10^−6^ mol/L) were added to a constant volume of 1 mL. The fluorescence spectra were obtained at an excitation wavelength of 360 nm.

### 2.5. Development of Paper-Based Sensor for Visual Detection of DA

The paper-based sensor containing the CdTe QDs solution (2.0 × 10^−6^ mol/L) was placed at 37°C and dried for 5–7 min. Rod-shaped nanoZnTPyP at a concentration of 3.055 × 10^−6^ mol/L was mixed with ultra-pure water (blank group), rod-shaped nanoZnTPyP was mixed with DA at different concentrations (experimental group). Subsequently, rod-shaped nanoZnTPyP (3.055 × 10^−6^ mol/L) and ultrapure water mixture (blank group), rod-shaped nanoZnTPyP and different concentrations of DA mixture (experiment group) were added to the paper sensor device respectively. The fluorescence color quenching and recovery results on the paper-based sensor were observed under the UV light of 365 nm, and a smartphone was used to take pictures and record. With the help of Photoshop software, the picture of color change on the paper device was carried out and RGB values were used to represent color information. The corresponding RGB value data was collected using MATLAB software, and the information matrix was established. Finally, the partial least squares discriminant analysis (PLSDA) model was used to quickly classify and analyze DA with different concentrations and different substrates.

## 3. Results and Discussion

### 3.1. Characterization of Rod-Shaped NanoZnTPyP and CdTe QDs

**Figure 1:**
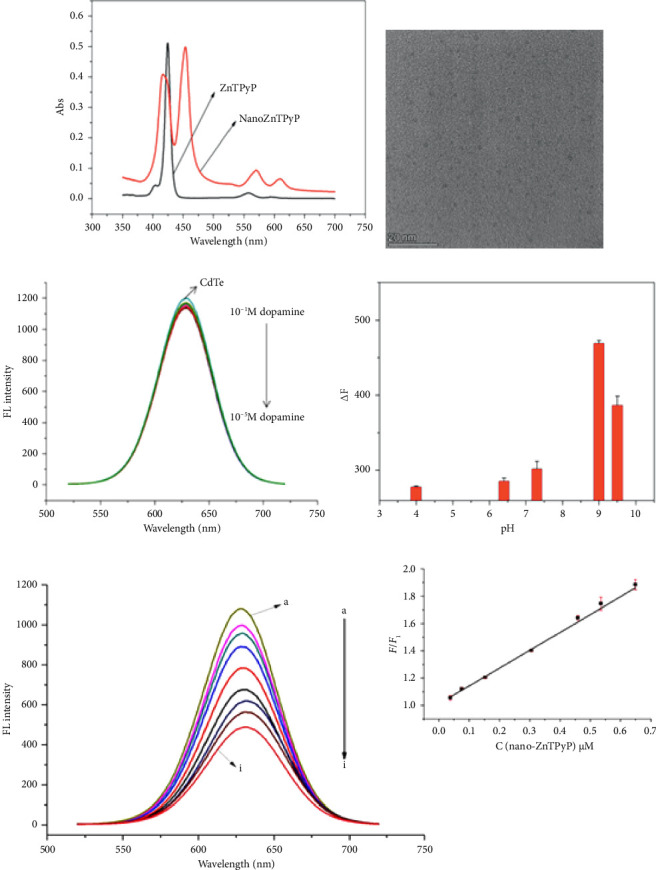
(a) UV absorption spectrum of ZnTPyP porphyrin and nanoZnTPyP; (b) TEM detection result of nanoZnTPyP; (c) fluorescence spectrum of CdTe quantum dots with the addition of different concentrations of dopamine; (d) effect of different pH on the detection of dopamine by fluorescence sensor; (e) the fluorescence intensity for CdTe quantum dots in presence of nanoZnTPyP (0.0382∼0.649 μmol/L); the inset: the fluorescence quenching linearity of CdTe QDs in the range of 0.0382∼0.649 μmol/L of nanoZnTPyP.

Compared with the absorption spectrum of ZnTPyP (Figure 1(a)), the UV absorption spectrum of nanoZnTPyP has obvious peak splitting and peak broadening, which indicates that the nanoZnTPyP is successfully synthesized [29]. In addition, the morphological characteristics of rod-shaped nanoZnTPyP were characterized by TEM. The results are shown in Figure 1(b). The rod-shaped nanoZnTPyP was in the form of nanorods with a particle size of 50 ± 5 nm. The morphology and size of CdTe QDs were characterized by TEM. The prepared CdTe QDs (Figure S2A) are spherical and have a diameter of about 4 nm.

### 3.2. Feasibility Analysis and Mechanism Investigation of Detecting DA

We examined the feasibility of this method. The fluorescence spectrum (Ex = 380 nm) recorded the results of different concentrations of DA directly acting on CdTe QDs (Figure 1(c)). The fluorescence intensity of CdTe QDs when detecting different concentrations of DA (1.0 × 10^−6^∼1.0 × 10^−8^ mol/L) was almost unchanged. The role of DA and CdTe-rod NanoZnTPyP was explored [30]. The UV absorption spectrum of nanoZnTPyP and the fluorescence emission spectrum of CdTe QDs overlapped at a wavelength of about 610 nm (Figure S2B). It was confirmed that nanoZnTPyP quenched CdTe QDs by FRET and PET. The results of UV-Vis spectra are shown in Figure S2C. After adding DA, the peaks at 420 and 450 nm showed a slight red shift, and UV absorption intensity at 420, 450, and 620 nm all decreased, indicating that DA combined with the prepared sensor and caused a structural change in the sensor. To further clarify the effect of DA and CdTe-rod nanoZnTPyP, the mid-infrared spectra of CdTe QDs, CdTe-rod nanoZnTPyP and CdTe-rod nanoZnTPyP after DA addition were studied (Figure S2D). The C-O stretching vibration is attributed to the carbonyl group in the CdTe QDs at 1050 and 1350 cm^−1^ is attributed to the −OH or −NH stretching vibration of the hydroxyl or amino functional group in the CdTe QDs [31, 32]. In addition, the absorption at 1680 cm^−1^ is attributed to C=O of CdTe QDs [33]. After adding nanoZnTPyp to CdTe QDs, the infrared spectra produced absorption peaks at 2750 and 1350 cm^−1^, respectively, which was caused by the change of the phenyl structure of the metal porphyrin ZnTPyP [34]. When DA was added to the CdTe-rod nanoZnTPyP, a significant wavelength blue shift occurred at 3400 cm^−1^, indicating that the amino group and carboxyl group of DA could interact with the carboxyl groups of the CdTe QDs [35]. After adding DA, the absorption peaks of nanoZnTPyP at 2750 and 1350 cm^−1^ also decreased and disappeared, indicating that DA interacts with Indicating that DA interacts with hydroxyl or amino groups as well as with metal porphyrin ZnTPyP. The above results indicate that CdTe-rod nanoZnTPyP may specifically interact with the amino and hydroxyl groups of the characteristic group of DA, which makes it possible to detect DA. The above results indicated that CdTe QDs may specifically interact with the amino and hydroxyl groups of DA's characteristic groups, making it possible to detect DA (Figure S1). We speculate that the detection mechanism may be the oxidation of DA into alkaline CdTe QDs-rod nanoZnTPyP solution to produce DA-quinine, and the recovery of fluorescence was caused by the electronic effect of DA-quinine and rod-shaped nanoZnTPyP [27]. The XPS of CdTe QDs and CdTe QDs interacting with nanoZnTPyP can be further demonstrated, as shown in Figure S3 XPS observations show that the surface of CdTe QDs is partially oxidized after interacting with nanoZnTPyP. To further explore the influence of the morphology of nanoZnTPyP on the detection specificity, we synthesized spherical nanoZnTPyP (Figure S4A) and compared the effect of rod-shaped nanoZnTPyP in detecting DA. As shown in Figure S4B, it was found that after adding DA, the CdTe QDs-rod nanoZnTPyP sensor can effectively restore the original quenched fluorescence, while the fluorescence of the CdTe QDs-spherical nanoZnTPyP sensor has no obvious recovery. We have concluded that the rod-shaped nanoZnTPyP can better bind to DA and achieve the purpose of detecting DA.

### 3.3. Analysis of DA by Fluorescence Sensor

We further optimized the experimental conditions of this method. The pH optimization results are shown in Figure 1(d). The results were best under alkaline conditions. Finally, a Tris-HCl buffer solution with pH = 9.0 was selected. Firstly, the “Turn-off” process was studied. As can be seen from the results in Figure 1(e), the fluorescence intensity has been reduced to varying degrees after adding different concentrations of rod-shaped nanoZnTPyP to the CdTe QDs solution. Rod-shaped nanoZnTPyP and CdTe QDs conform to the “Stern-Volmer” equation: *F*_0_/*F*_1_ = 1 + Ksv t_0_ [M] when the concentration range of added rod-shaped nanoZnTPyP is 0.0382∼0.649 μmol/L. Where *F*_0_ is the original fluorescence, *F*_1_ is the fluorescence intensity of the QDs after adding rod-shaped nanoZnTPyP, [M] is the concentration of the added rod-shaped nanoZnTPyP, and Ksv is the quenching efficiency of rod-shaped nanoZnTPyP. The concentration of rod-shaped nanoZnTPyP was proportional to *F*_0_/*F*_1_ [25]. Rod-shaped nanoZnTPyP has a linear relationship with *F*_0_/*F*_1_ in the concentration range of 0.0382∼0.649 μmol/L (*R*^2^ = 0.995). The quenching of CdTe QDs by rod-shaped nanoZnTPyP has laid a good foundation for the subsequent recovery process.

**Figure 2:**
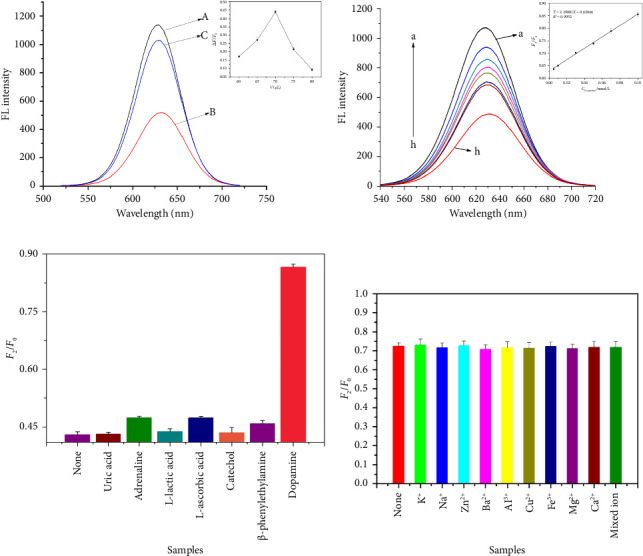
(a) The fluorescence recovery of CdTe QDs and nanoZnTPyP systems in the presence of dopamine (1 × 10^−7^ mol/L). Inset: the optimizing of the addition amount of nanoZnTPyP (0.459–0.612 μmol/L) during recovery of QDs fluorescence after the addition of Dopamine. (b) The recovery of CdTe QDs fluorescence in CdTe-nanoZnTPyP system with dopamine concentration range of 5.0 × 10^−10^∼1.0 × 10^−8^ mol/L; the inset: fluorescence recovery linearity of CdTe-nanoZnTPyP in presence of different concentration dopamine in range of 5.0 × 10^−10^∼1.0 × 10^−8^ mol/L. (c) The F2/F0 value of CdTe-rod nanoZnTPyP system when different common dopamine disruptors are added. (d) The F2/F0 value of CdTe-rod nanoZnTPyP system when different metal ions are added.

At the optimal pH conditions, the amount of rod-shaped nanoZnTPyP added was optimized (Figure 2(a)). The optimal volume of adding rod-shaped nanoZnTPyP was 70 μL, and the corresponding concentration of rod-shaped nanoZnTPyP was 0.535 μmol/L. Based on the above studies, this experiment further studied the fluorescence recovery process of CdTe-rod nanoZnTPyP after adding different concentrations of DA, where the concentration of DA was 5.0 × 10^−10^∼1.0 × 10^−8^ mol/L. The experimental results are shown in Figure 2(b). In this concentration range, DA was directly proportional to *F*_2_/*F*_0_ (*Y* = 2.29881*X* + 0.62846, *R*^2^ = 0.995), the limit of detection (LOD) is 0.38 nmol/L, the calculation of LOD was described in SI. The results indicated that DA had a good recovery effect on the CdTe-rod nanoZnTPyP system.

### 3.4. Specificity and Selectivity of Fluorescent Sensor

To evaluate the specificity of this method, uric acid, epinephrine, L-lactic acid, L-AA and its structural analogs catechol and β-phenylethylamine were studied. They are common interfering substances of DA in human plasma [36]. The concentration of interfering substances was 1.0 × 10^−6^ mol/L. The fluorescence recovery phenomenon (Figure 2(c)) of the CdTe-rod nanoZnTPyP system was generated only by adding DA (1.0 × 10^−8^ mol/L). It is worth noting that although the electrochemical properties of uric acid and L-AA are very similar to DA, they did not affect CdTe-rod nanoZnTPyP sensor system used to detect DA. The results have clearly demonstrated the high selectivity of the fluorescence sensor system developed for DA.

In addition, the interference of other metal ions on the method was also studied. The metal ions (K^+^, Na^+^, Zn^2+^, Ba^2+^, Al^3+^, Cu^2+^, Fe^3+^, Mg^2+^, Ca^2+^, and mixed ions) with a concentration of 1.0 × 10^−5^ mol/L were selected and added to the mixed solution. As shown in Figure 2(d), these metal ions have not caused significant changes in fluorescence intensity in the past. It has been further explained that this method has excellent selectivity.

### 3.5. Analysis of Actual Samples

The CdTe-rod nanoZnTPyP colorimetric sensor has shown excellent performance for DA detection. To prove that the CdTe-rod nanoZnTPyP sensor can be used for the analysis of actual samples, we selected human plasma as the complex matrix for detecting DA. The recovery rate was calculated by adding a known amount of DA. The results in Table 1 are shown that the recovery of DA in human plasma was 96.5%∼99.1%, and the precision RSD was 3.8%∼5.9% (*n* = 3). Simultaneously, the results were compared with the HPLC results (Table S1). It has been shown that the CdTe-rod nanoZnTPyP fluorescence sensor can be used for the detection of DA in human plasma. It has been shown that the sensor has potential application value in the actual detection of DA.

### 3.6. Paper-Based Sensor Detection of DA Analysis Process

We found that this “turn-off-on” mode is accompanied by a corresponding change in fluorescence color. Therefore, the combination of colorimetric sensor and paper sensor technology can achieve the purpose of rapid colorimetric detection of DA [37].  Figure S5 is shown the results of DA detection by paper-based sensor colorimetry. After adding rod-shaped nanoZnTPyP, the fluorescence of CdTe QDs quenched and showed blue-violet. The color of the paper-based sensor device substrate was restored to the original rose red color when the mixed solution of DA and rod-shaped nanoZnTPyP was added to the paper-based sensor CdTe QDs substrate.

**Figure 3:**
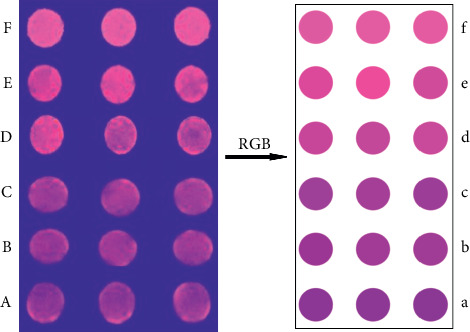
Colorimetric changes for dopamine samples with different concentrations (A∼F corresponding to 1, 10, 50, 100, 500, 1000 nmol/L, respectively) based on CdTe-nanoZnTPyP paper-based sensor.

**Figure 4:**
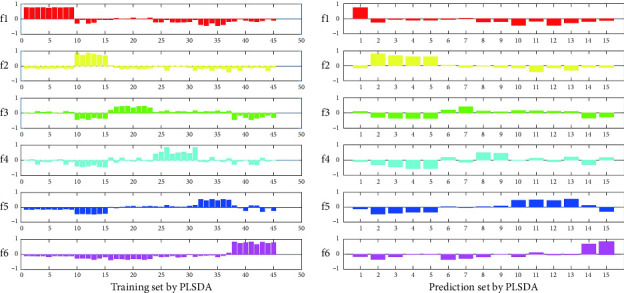
Assigned plots of dummy codes of training and prediction set with the different concentration dopamine based on CdTe-nanoZnTPyP sensing in PLSDA model.

To further evaluate the detection performance of this method, we used paper-based sensor technology to detect DA based on the CdTe-rod nanoZnTPyP sensor system.  Figure 3(A–F) corresponds to the generation of DA at concentrations of 1, 10, 50, 100, 500, and 1000 nmol/L, respectively. The color of the paper-based sensor changed from the original dark purple to light purple and purplish-red, with the increase of DA concentration. At a concentration of 1000 nmol/L, it is close to the original fluorescent rose red color of CdTe QDs. The color response on the paper device of each density was measured 10 times in parallel. When the DA concentrations were 1, 10, 50, 100, 500, and 1000 nmol·L^−1^, the RGB value information of 60 samples were obtained. Then randomly divide it into a training set and a prediction set. The samples have been pattern-recognized according to the position of the largest virtual code. The recognition rates of the training set and prediction set were 100% (Figure 4). The attribution of the samples obtained by the PLSDA model is shown in Table S2. The results have shown that the paper-based sensor can detect different concentrations of DA to produce a clear color response, and has good sensitivity. It has been shown that the CdTe-rod nanoZnTPyP fluorescent visualization paper-based sensor and the PLSDA model provides a reliable method for the visual detection and analysis of DA.

In the actual analysis, the rapid and immediate detection method, instrument-free one-site detection has become one of the problems that need to be solved urgently. Therefore, we have further developed and applied it to paper-based sensor, which can achieve simple operation, sensitive and instant instrument-free detection. As shown in Figure S6, complex matrix samples (human plasma) containing different concentrations of DA (1–1000 nmol L^−1^) mixed with nanoporphyrins were added dropwise on paper sensor substrates prepared with CdTe QDs, resulting in significantly different degrees of color recovery. In order to accurately identify the different concentrations of DA in a complex matrix, we used MATLAB software to convert the images in the actual sample into RGB color values, and constructed a data matrix. Then, we used a PLSDA model to discriminate different concentrations of DA in actual sample. We conducted a discriminant analysis of DA in human plasma. There were 6 different concentrations of DA in human plasma matrix (1–1000 nmol/L), with 10 samples at each concentration, giving a total of 60 samples. 60 samples were randomly divided into training sets and prediction sets (Table S3). The virtual codes of the 6 concentrations of DA samples were f1, f2, f3, f4, f5, and f6. The PLSDA discrimination results are shown in Figure S7. Different concentrations of DA e in human plasma reached a recognition rate of 100% in the training set and the prediction set. Both sensitivity and specificity were 1. The results have shown that the paper-based sensor and PLSDA model can discriminate the concentrations of DA in human plasma. Furthermore, we used the PLSR model to correlate the RGB values of the paper-based sensor with the concentration of DA in human plasma (Figure S8). We obtained a low root-mean-square errors (RMSEs) through eight-fold cross-validation to determine the hidden variables (LVs). The best LVs for the PLSR model in human plasma were 9. The corrected RMSE (RMSEC) and the predicted RMSE (RMSEP) are used to evaluate the accuracy of the corrected model. The predicted results of DA concentration in human plasma are shown in Table S4. The correlation coefficients between actual and predicted concentrations of the training and prediction sets were greater than 0.9999 (Rc) and 0.9941 (Rp), respectively, and the RMSE did not exceed 0.0381. The results have shown that the RGB value of the paper-based sensor combined with the PLSR model can be used to predict the concentration of DA in human plasma, and can provide an accurate and reliable quantitative result.  Table 2 compares various analytical methods that have been reported to detect DA. This method has a relatively low detection limit and can visually detect the content of DA in human plasma. The sensor has been verified to have potential application for the detection of DA in complex samples.

## 4. Conclusion

In this paper, a new method of DA analysis based on CdTe-rod nanoZnTPyP fluorescence paper-based sensor is designed and developed. Utilizing the characteristics of rod-shaped ZnTPyP NPs that can better bind to the target, combined with the advantages of the QDs “Turn-off-on” model, high selectivity and high sensitivity detection of DA has been achieved. More importantly, the paper-based sensor is based on the different color intervals formed by the response of CdTe-rod nanoZnTPyP system to different concentrations of DA, which realized its fast and accurate visual analysis. The paper-based sensor has a simple preparation process and rapid color development. It provides an efficient, convenient, reliable and rapid method and new ideas for one-site detection of DA.

## Figures and Tables

**Table 1:** Detection of dopamine in human plasma samples.

Samples	Add (nmol/L)	Fund (nmol/L)	Recovery (%)	RSD (%) (*n* = 3)
Human plasma	10	9.75	96.5	3.8
	5	5.97	99.4	4.1
	1	0.99	99.1	5.9

**Table 2:** Comparison of this method with the reported literature for the detection of dopamine.

Analytical methods	Material	Linear range (nmol/L)	LOD (nmol/L)	References
HPLC-UV	—	0.39–163.19	0.13	[5]
Electrochemical method	TCPP/CCG	100∼1000	22	[38]
Chemiluminescence	Lucigenin−RF	5.6∼55.6 × 10^3^	1.87	[39]
Colorimetry	Au NPs	10∼1000	46	[8]
Fluorescence analysis	CDZs	180∼1.5 × 10^4^	1.06	[14]
Fluorescence analysis	Polydopamine	1.0 × 10^3^∼2.0 × 10^5^	300	[31]
Fluorescence analysis	CdTe-nanoZnTPyP	0.5∼10	0.38	This work

## Data Availability

The data that support the findings of this study are available from the corresponding author upon reasonable request.

## Supporting Information

Figure S1: Schematic illustration of the developed CdTe QDs-based fluorescent probe for dopamine.

Figure S2: (A) TEM detection result of CdTe QDs; (B) The UV absorption spectra of nanoZnTPyP and the fluorescence emission spectra of CdTe QDs; (C) UV absorbance spectrum of CdTe, CdTe-nanoZnTPyP, CdTe-nanoZnTPyP + dopamine and dopamine; (D) Mid-infrared spectrum of dopamine detection based on CdTe, CdTe + nanoZnTPyP and CdTe-nanoZnTPyP + dopamine.

Figure S3: XPS of O 1 s (a), Te 3 d (b), Cd 3 d (c) and S (d) in CdTe, CdTe + nanoZnTPyP and CdTe-nanoZnTPyP + dopamine.

Figure S4: (A) TEM detection result of spherical nanoZnTPyP; (B) Fluorescence recovery of CdTe-spherical nanoZnTPyP and CdTe-rod nanoZnTPyP after reaction with dopamine.

Figure S5: Feasibility analysis of detection of dopamine based on CdTe QDs and NanoZnTPyP paper-based sensor.

Figure S6: Visual detection of different concentrations (A∼F corresponding to 1, 10, 50, 100, 500, 1000 nmol/L, respectively) of dopamine in actual samples (human plasma) based on paper-based sensor.

Figure S7: Assigned plots of dummy codes of training and prediction set with the different concentration dopamine in human plasma based on CdTe-NanoZnTPyP sensing in PLSDA model.

Figure S8: Correlation curve between actual concentration and predicted concentration of dopamine in human plasma based on RGB value of paper-based sensor.

Table S1: Detection of Dopamine in Human Plasma by HPLC Method.

Table S2: A detailed splitting information of training set and prediction set of RGB data measured by different concentrations of dopamine based on PLSDA model.

Table S3: A detailed splitting information of dopamine in human plasma with different concentrations (1–1000 μmol/L).

Table S4: Results for concentrations of dopamine in human plasma based on PLSR models.
