# Supplementary material for: Rod-Shaped NanoZnTPyP Paper-Based Sensor for Visual Detection of Dopamine in Human Plasma
Source: J Anal Methods Chem. 2025 Apr 14;2025:9981628. doi: 10.1155/jamc/9981628 (PMC12011473; doi:10.1155/jamc/9981628)
Supplement: Supporting Information — Additional supporting information can be found online in the Supporting Information section. [file 9981628.f1.docx]

## Support Information

**Rod-shaped nanoZnTPyP paper-based sensor for visual detection of dopamine in human plasma**

**Linlin Yin^a^*, Yuyu Du^b^, Miaohua Ge^a^,Xiang Zhang^a^,** **Xinyi Du^a^, Xiaoqiong Wu^a^***

^a^ *Jiaxing Center for Disease Control and Prevention, Jiaxing 314050, PR China*

^b^ *Jiaxing Jiayuan Testing Technology Service Co., Ltd, Jiaxing 310032, PR China*

## Corresponding Author

^*^ Xiaoqiong Wu, +86-0573-83685809, fax: +86-0573-83685809

E-mail: 285016065@qq.com

^*^Linlin Yin, +86-0573-83683808, fax: +86-0573-83683808

E-mail: ylinrr@163.com

LOD=3.3 × (standard deviation/slope)=3.3 × (0.265/2.29881)


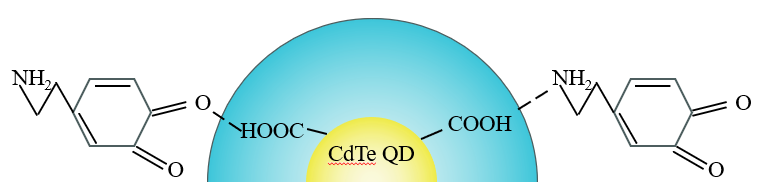
Fig. S1 Schematic illustration of the developed CdTe QDs-based fluorescent probe for dopamine.


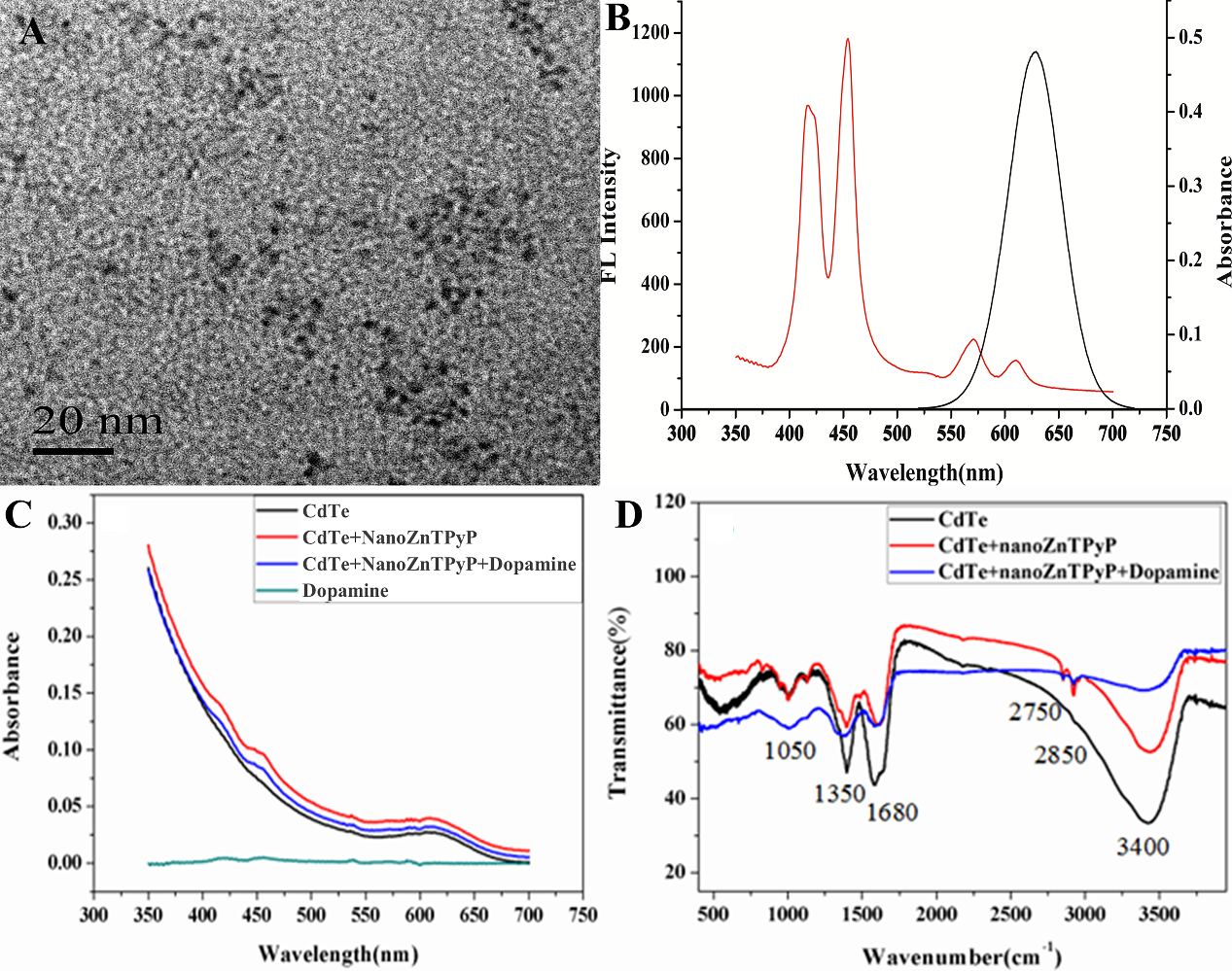


Fig. S2 (A) TEM detection result of CdTe QDs; (B) The UV absorption spectra of nanoZnTPyP and the fluorescence emission spectra of CdTe QDs; (C) UV absorbance spectrum of CdTe, CdTe-nanoZnTPyP, CdTe-nanoZnTPyP + dopamine and dopamine; (D) Mid-infrared spectrum of dopamine detection based on CdTe, CdTe + nanoZnTPyP and CdTe-nanoZnTPyP + dopamine.


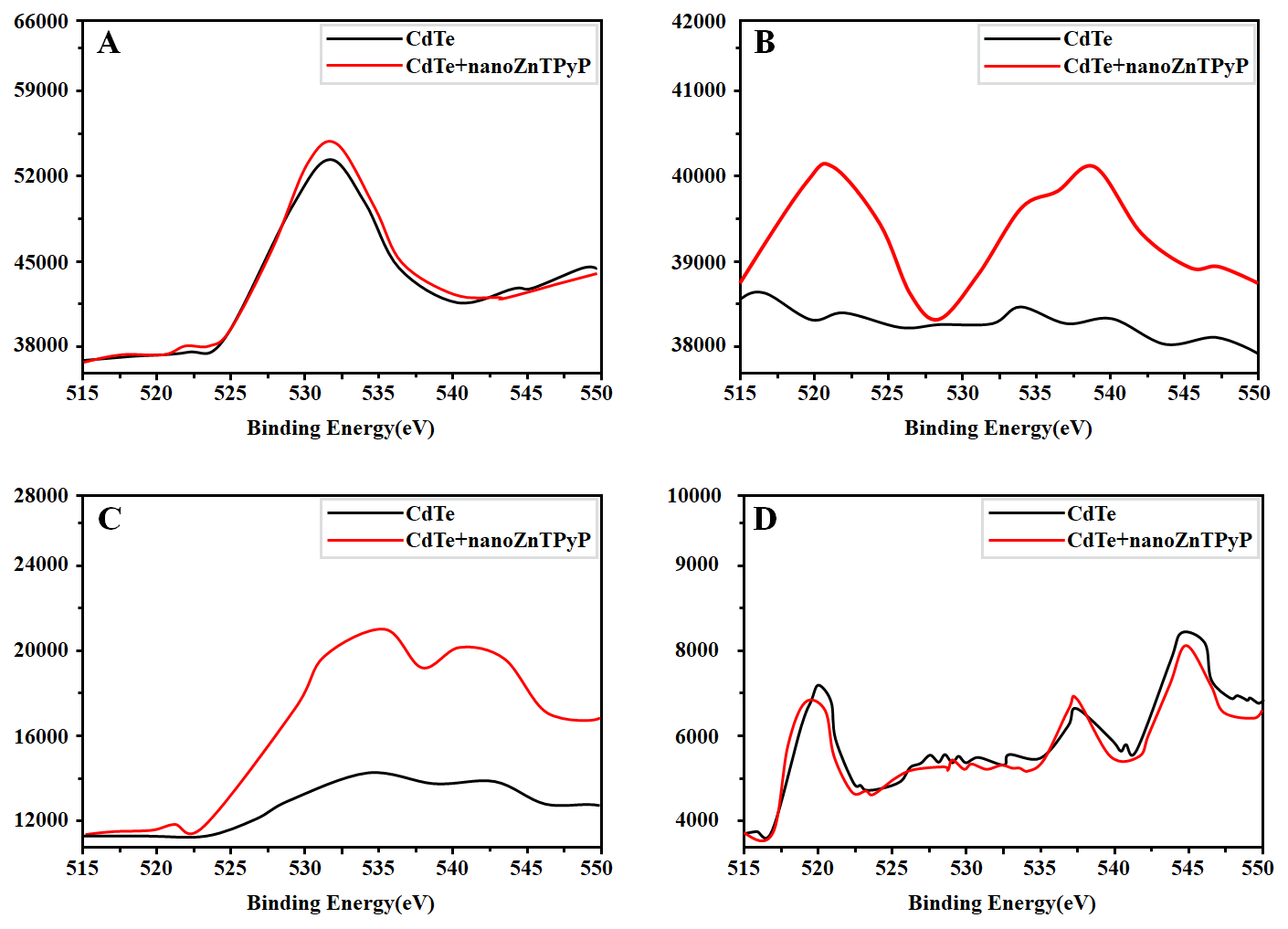


Fig. S3 XPS of O 1s (a), Te 3d (b), Cd 3d (c) and S (d) in CdTe, CdTe + nanoZnTPyP and CdTe-nanoZnTPyP + dopamine.


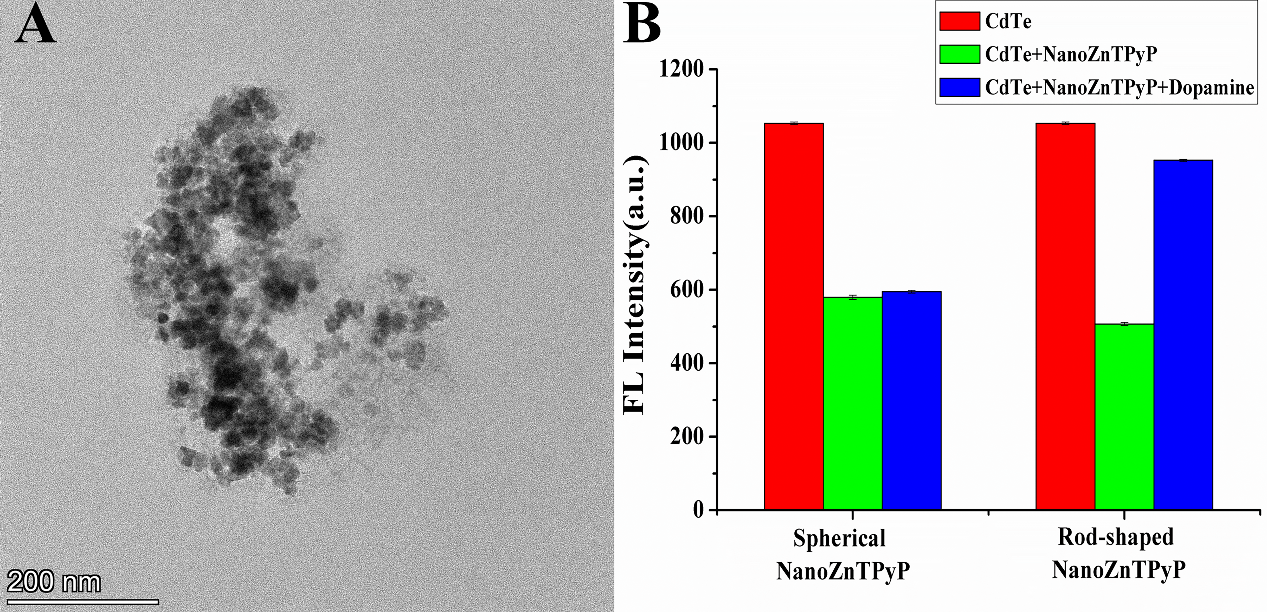


Fig. S4 (A) TEM detection result of spherical nanoZnTPyP; (B) Fluorescence recovery of CdTe-spherical nanoZnTPyP and CdTe-rod nanoZnTPyP after reaction with dopamine.


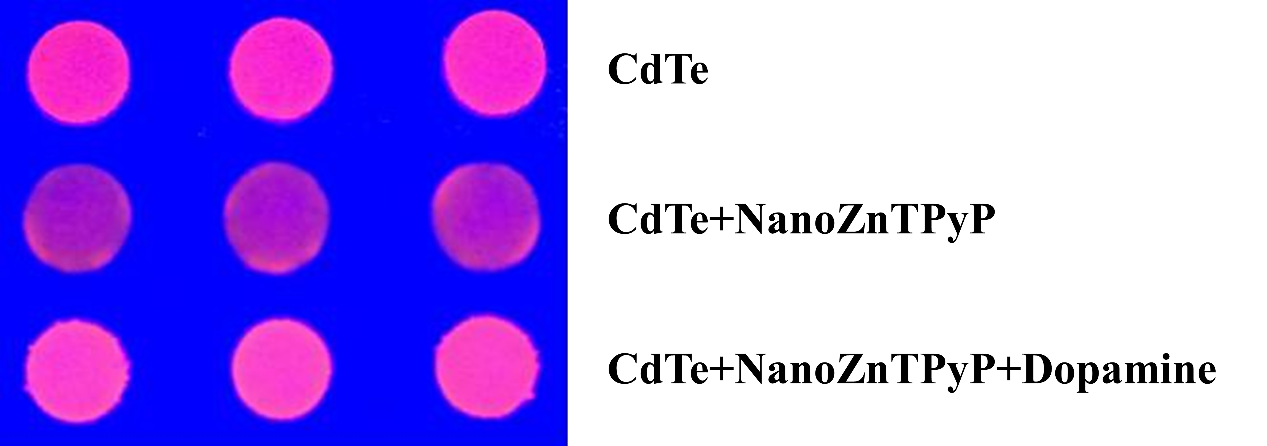


Fig. S5 Feasibility analysis of detection of dopamine based on CdTe QDs and NanoZnTPyP paper-based sensor.


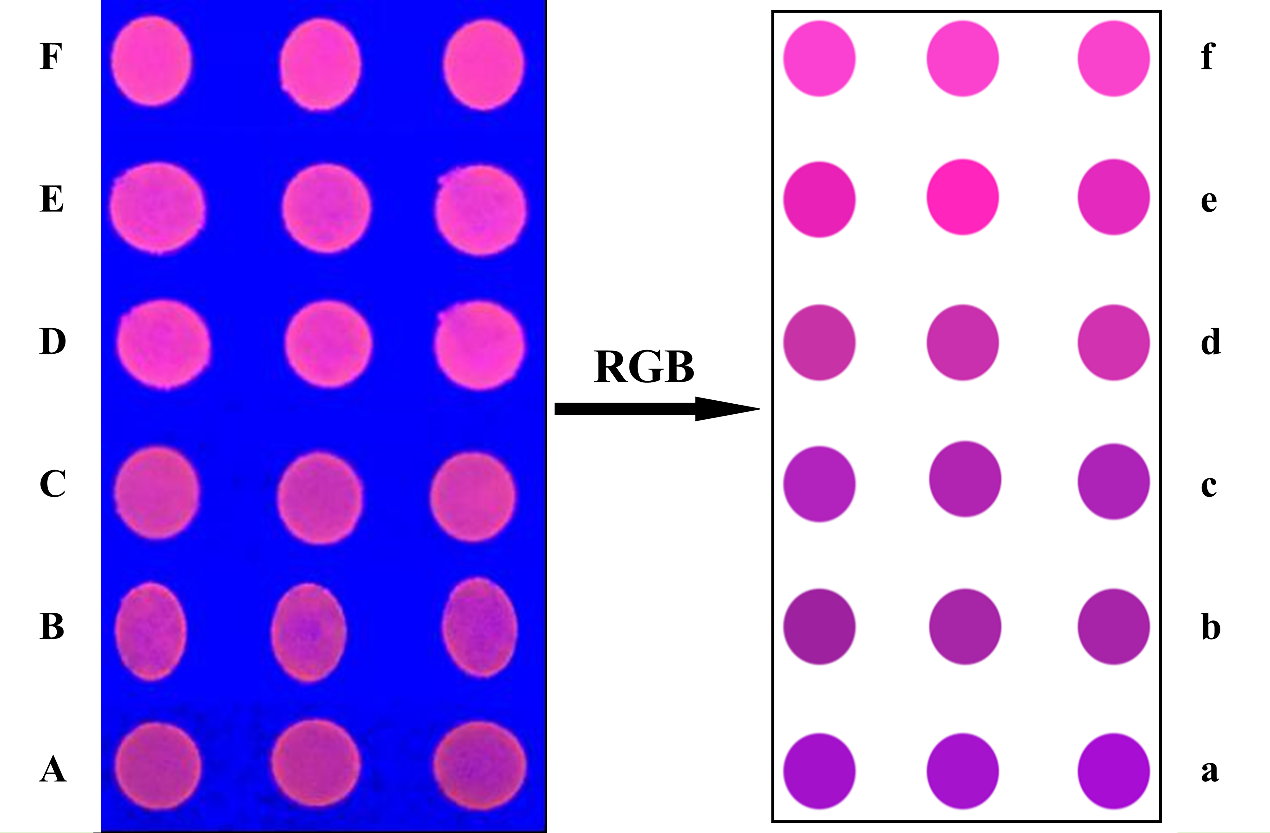


Fig. S6 Visual detection of different concentrations (A~F corresponding to 1 nmol/L, 10 nmol/L, 50 nmol/L, 100 nmol/L, 500 nmol/L, 1000 nmol/L respectively) of dopamine in actual samples (human plasma) based on paper-based sensor.


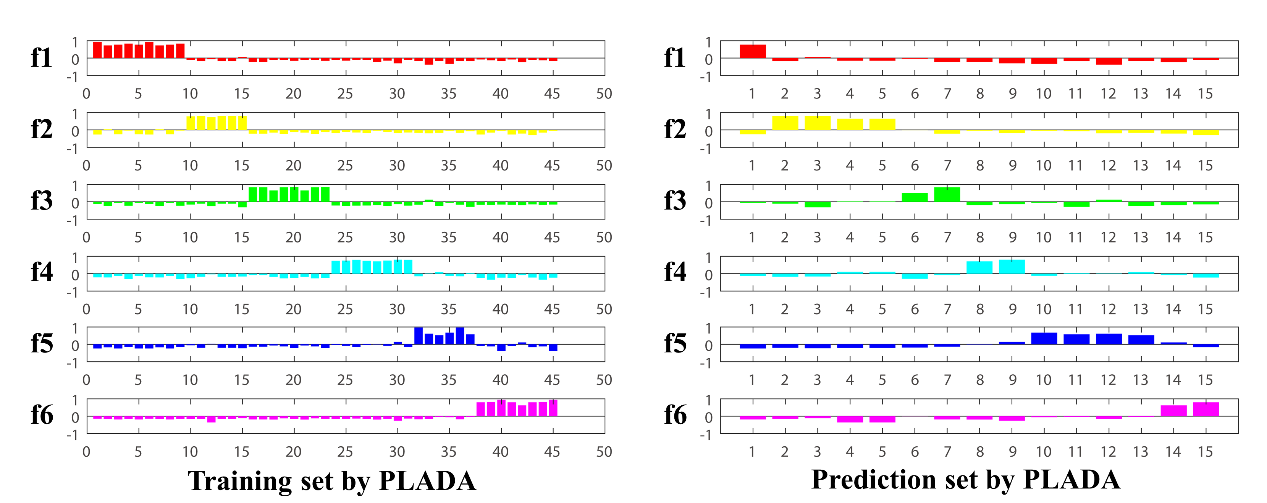


Fig. S7 Assigned plots of dummy codes of training and prediction set with the different concentration dopamine in human plasma based on CdTe-NanoZnTPyP sensing in PLSDA model.


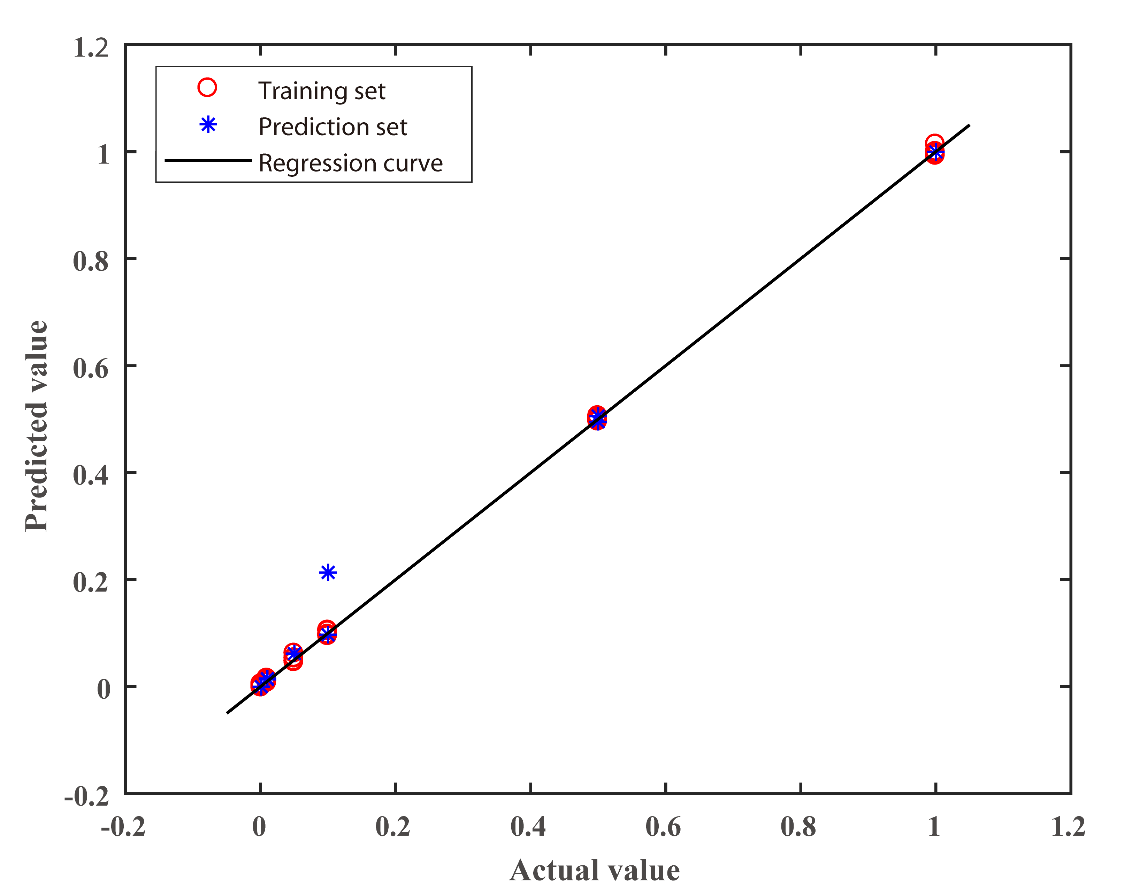


Fig. S8 Correlation curve between actual concentration and predicted concentration of dopamine in human plasma based on RGB value of paper-based sensor.

Table.S1 Detection of Dopamine in Human Plasma by HPLC Method.

| Samples | Add (nmol/L) | Fund (nmol/L) | Recovery (%) | RSD (%) (n=3) |
| --- | --- | --- | --- | --- |
| Human plasma | 10  5  1 | 10.12  4.94  1.02 | 101.2  98.8  102.0 | 1.2  2.7  4.6 |

Table.S2 A detailed splitting information of training set and prediction set of RGB data measured by different concentrations of dopamine based on PLSDA model.

| Group code | Training set | | Prediction set | |
| --- | --- | --- | --- | --- |
|  | Number | Samples | number | samples |
| 1000  500  100  50  10  1 | 9  6  8  8  6  8 | 1^st^-9^th^  10^th^-15^th^  16^th^-23^rd^  24^th^-31^st^  32^nd^-37^th^  38^th^-45^th^ | 1  4  2  2  4  2 | 1^st^  2^nd^-5^th^  6^th^-7^th^  8^th^-9^th^  10^th^-13^rd^  14^th^-15^th^ |

Table.S3 A detailed splitting information of dopamine in human plasma with different concentrations (1-1000 μmol/L).

| Group code | Training set | | Prediction set | |
| --- | --- | --- | --- | --- |
|  | Number | Samples | number | samples |
| f1  f2  f3  f4  f5  f6 | 9  6  8  8  6  8 | 1^st^-9^th^  10^th^ -15^th^  16^th^-23^rd^  24^th^-31^st^  32^nd^-37^th^  38^th^-45^th^ | 1  4  2  2  4  2 | 1^st^  2^nd^-5^th^  6^th^-7^th^  8^th^-9^th^  10^th^-13^rd^  14^th^-15^th^ |

Table. S4 Results for concentrations of dopamine in human plasma based on PLSR models.

| Matrices | Rc | RSMEC | Rp | RSMEP |
| --- | --- | --- | --- | --- |
| Human plasma | 0.9999 | 0.0046 | 0.9941 | 0.0381 |
